# A teaching and training framework to promote findable, accessible, interoperable, and reusable data generation in agriculture

**DOI:** 10.1093/database/baaf034

**Published:** 2025-04-25

**Authors:** Annarita Marrano, Leyla Cabugos, Alenka Hafner, Beant Kapoor, John McNamara, Megan O’Donnell, Leonore Reiser, Marcela Karey Tello-Ruiz, Huiting Zhang, Margaret Staton

**Affiliations:** Phoenix Bioinformatics, 39899 Balentine, Dr #200, Newark, CA 94560, United States; Robert E. Kennedy Library, California Polytechnic State University, 1 Grand Ave, Bldg. 35, San Luis Obispo, CA 93407, United States; Department of Biology, Pennsylvania State University, 361 Frear North Building, 152 Old Coaly Way, University Park, State College, PA 16802, United States; Department of Entomology and Plant Pathology, University of Tennessee, 2505 EJ Chapman Drive, 370 PBB, Knoxville, TN 37996, United States; Department of Animal Science, Washington State University, PO Box 646310, Pullman, WA 99164-6310, United States; University Library Research Data Services, Iowa State University, 204 Parks Library, 701 Morrill Rd, Ames, IA 50011, United States; Phoenix Bioinformatics, 39899 Balentine, Dr #200, Newark, CA 94560, United States; Cold Spring Harbor Laboratory, 1 Bungtown Rd, Cold Spring Harbor, Laurel Hollow, NY 11724, United States; Department of Horticulture, Washington State University, 249 Clark Hall, PO Box 646414, Pullman, WA 99164-6414, United States; Department of Entomology and Plant Pathology, University of Tennessee, 2505 EJ Chapman Drive, 370 PBB, Knoxville, TN 37996, United States

## Abstract

Advances in agricultural genetic, genomic, and breeding (GGB) technologies generate increasingly large and complex datasets that need to be adequately managed and shared. While several agricultural biological databases maintain and curate GGB data, not all scientists are aware of them and how they can be used to access and share data. In addition, there is the need to increase scientists’ awareness that appropriate data archiving and curation increases data longevity and value and bolsters scientific discoveries’ reproducibility and transparency. The AgBioData Education working group aims to address these unmet needs and developed a modular curriculum for educators teaching the basics of biological databases and the findable, accessible, interoperable, and reusable (FAIR) principles to undergraduate and graduate students (https://www.agbiodata.org/). The present paper provides an overview of the topics covered within the curriculum, called ‘AgBioData Curriculum for Ag FAIR Data,’ its audience and modalities, and how it will positively impact all the different stakeholders of the agricultural database ecosystem. We hope the modular curriculum presented here can help scientists and students understand and support database use in all aspects of improving our global food system.

**Database URL**: https://zenodo.org/records/14278084

## Introduction

Generating larger and more complex genetic, genomic, and breeding (GGB) data has heightened the need for guidelines for agricultural research data sharing and management. Data correctly stored and described in public databases and repositories can be repurposed to fuel new discoveries and verify current science. Appropriate data archiving and curation increases data longevity and value, which in turn bolsters the reproducibility and transparency of scientific discoveries [1]. While many biological databases are dedicated to agricultural research, not all scientists know how they can be used to find and share data. Therefore, there is a strong need to educate current and future GGB researchers about the role and importance of databases and data repositories in agricultural science and how to make their data findable, accessible, interoperable, and reusable (FAIR) [2].

The AgBioData consortium, which involves over 40 GGB databases and associated resources, was formed to engage database repository leaders, developers, curators, users, funders, and publishers to improve existing and create new guidelines to support the implementation of FAIR data standards in agricultural research [3]. In addition to advancing GGB data accessibility, interoperability, reusability, and sustainability, one of the core aims of the consortium is to provide an educational framework about data repositories, and GGBs in particular, in the storage, curation, and dissemination of FAIR biological data. Education on the importance and use of databases will help advance agricultural science rapidly and sustainably by equipping scientists and other stakeholders to use public data and understand how to share their own data effectively. In 2021, AgBioData established the volunteer Education Working Group (EWG) to discuss and address the gaps in GGB database education. The EWG involves graduate students, professors, librarians, and database curators (https://www.agbiodata.org/working_groups/education) with experience or interest in education and FAIR data management.

Since their introduction in 2016, the FAIR principles have garnered support across science disciplines [4, 5], leading to several outreach initiatives on FAIR data management and stewardship [6, 7]. However, most of these educational materials are not framed for students new to both their field and FAIR data. In addition, there are few educational curricula on GGB community databases and their crucial role in FAIR agricultural data. While database technologies and data management are often included in computer science majors, they are frequently missing in biological science disciplines. Yet nearly every biological scientist uses data repositories in their research. Activities include archiving data and the associated descriptive information (metadata) [8]; searching, accessing, and downloading data; analysing raw data using software or pipelines implemented in a database [9]; looking for publications or training material; and connecting with the scientific community.

Consequently, many scientists must learn independently to navigate an ever-expanding array of databases, choose which ones to use, and self-train on how and what to share. With the increasing number of data types and databases available, it can be overwhelming for scientists to know where to submit their data or how [10, 11]. When scientists share data, they often do so in non-standard formats and with poorly described metadata [12]. Educating scholars about databases, how to use them, and the importance of making data FAIR would help current and future research students and professionals navigate the database ecosystem and encourage them to follow the FAIR guidelines. The need for explicit training material on FAIR data is even more pressing following the demand by many funding agencies worldwide (e.g. the European Research Council [13] and the US Office of Science and Technology Policy [14] for data management plans (DMPs) in grant proposals and for the resultant research data to be publicly and equitably accessible.

In addition to the need for educational material on databases for students, there is a need for teaching support for faculty who often carry the burden of preparing classes on topics with which they need to become more familiar [15]. An open-access curriculum with flexible modules, defined learning outcomes, and suggested activities to engage the students in the learning process would be a helpful resource for mentors who can decide to use the curriculum as it is or select and adapt lesson plans in their classroom. Further, the availability of open-access learning modules that are kept updated as the database community and standards evolve would lower access barriers in higher education due to technology and resource inequalities, thus promoting inclusion and equity in the database learning ecosystem [16].

These needs are reflected in the AgBioData membership, which the EWG queried about the need for training material for students and database users as part of a broader AgBioData survey conducted in 2022 to assess the awareness and implementation of the FAIR principles in GGB databases (survey results available at https://www.agbiodata.org/bsurvey-stakeh). While 84% of the survey participants agree that ‘GGB databases related to their work provide useful resources for learning how to use them (e.g. tutorials, FAQs, etc.)’, of the roughly 50% of respondents who had some experience submitting data to a GGB database, 18% reported it was not easy to do so in a satisfactory way, and a further. In comparison, 4% reported they found it very difficult or impossible. There was strong interest in prioritizing the creation of additional training materials for GGB database use and data submission, as well as specifically for formal educational materials about FAIR data management and best practices for undergraduate (UG) and graduate courses. One participant noted, ‘educational materials would be immensely useful so instructors don’t have to all write their own guides from scratch … many of these databases are underutilized because principal investigators assume their graduate students either know how to use them or will figure it all out on their own. The result is a community of users not entirely comfortable with the platforms or secure in their understanding of what the data means’. A prominent thread in responses across respondent groups (research scientists, bioinformatics professionals, postdoctoral scholars, and graduate students) was a desire for learning modalities to be varied and complementary, for instance, pairing static/asynchronous online tutorials (the most frequently mentioned format) with live workshops and/or short videos. There was also some interest in tailored help, such as through online forums, Q + A documents, or ‘office hours’ with trainers. Several graduate students requested that the training feature examples of use cases.

In summary, despite the routine use of biological databases in agricultural research and education, training materials on FAIR data management specifically for agricultural data are still missing. The EWG aimed to address this unmet need and developed a modular curriculum called ‘AgBioData curriculum for FAIR Ag Science’, targeting primarily educators teaching UG and graduate students how to search, download, analyse, and submit research data, work with databases, or curate data according to the FAIR principles. In this white paper, we present the curriculum, its design, and how it will positively impact the different stakeholders of the database ecosystem in agricultural research, emphasizing the crucial role of GGB community databases.

## The AgBioData curriculum for Ag FAIR data

We designed the curriculum considering the needs of current and future database users, as well as the basic knowledge about data management required for them to share and curate data. The curriculum includes seven lesson modules listed in Table 1 and was designed around core concepts and specific learning objectives needed to use databases following the FAIR principles in biology and agricultural research. The experience of the EWG members as database users, students, biocurators, educators, and librarians contributed the most to the choice of the lesson plans’ learning outcomes. Hereafter, we provide some insights into the curriculum content and its three core goals: (a) facilitate the choice and use of biological database resources, (b) understand the FAIR data principles and how they are applied to agricultural data management, and (c) empower GGB community databases in agricultural research. We also highlight how the AgBioData Curriculum for FAIR Ag Data will positively impact all stakeholders of the biological database ecosystem.

**Table 1.** List of the lesson modules of the presented curriculum, with a per-lesson summary of the topics covered and the target audience

Module	Module title	Topics covered	Learning outcomes	Target
LP1	What is a biological digital repository?	Brief history of databases; when and why first databases were established Overview of the variety of terms used to refer to databases and the differences among them Description of the spectrum of scientific databases Introduction to the relationship between the different categories of data and their stakeholders	Can explain the terms digital repository, database, web portal, website, resource, data repository, data bank, archive, library, and their overlap Can explain why databases were originally developed and how they support the research community today Can explain the spectrum of scientific databases from large, general-purpose to targeted, community-specific Can explain and discuss the relationships among the terms open data, primary data, secondary data, metadata, data provenance, data management, data curation Can explain the relationship between data producers, data users, open data, and technology	HS, UG, Grad
LP2	FAIR and databases	Overview of the FAIR principles, with examples in biological science	Can paraphrase the acronym FAIR Can explain why the FAIR principles are important and when they have been introduced Can give a general overview of the FAIR principles, explaining what digital objects they apply to, their history, and why they have been introduced Can describe the guidelines for making a digital object findable Can explain what a persistent identifier is and why it is important Can define the guidelines related to the ‘accessible’ principles Can explain when an object is interoperable and what are the most common conserved vocabularies used by biological databases Can explain how biological databases relate to FAIR guidelines Can paraphrase the CARE principles, describe them, and explain why they complement the FAIR principles	HS, UG, Grad
LP3	Bio-Databases: types of data, finding and obtaining data	Introduction to the diversity of data types managed in biological databases Biocuration: what it is and why it is important How to define an effective search query when looking for specific data Overview of the different mechanisms of transferring and obtaining data from a database	Understand the diversity of data types archived and managed in biological databases Learn what biocuration is and why it is important Learn how and where to find data Learn how to obtain data using graphical user interface and command line interface tools	HS, UG, Grad
LP4	Creating and sharing trustworthy data	Overview of the TRUST principles and why they are important Data Management Plan and how to write it	Understand that trust is a core part of data and database appraisal Articulate how good data management ensures good and trustworthy data sharing	UG, Grad
LP5	Submitting data	The importance of submitting data in a way that are accessible and reusable to the large community Overview of licenses and public policies	Understand the importance of submitting the generated data to biological digital repositories Learn to identify appropriate repositories for their data types and how to work with repositories to ensure data submission Learn how to prepare data for submission and where to submit them	UG, Grad
LP6	How to use your library resources	How to identify data services and curated research guides available at your institutions How to access scholarly literature through your institution or as an independent researcher	Identify data services available at academic institutions or others Find subject specialist librarians and curated research guides at academic institutions or, if not available, at other institutions Access scholarly literature through your institution or as an independent researcher	UG, Grad
LP7	Databases for agriculture	Where agricultural data are submitted How GGB databases facilitate discoveries and progress in agriculture	Can understand and list which data are generated and used in agricultural research Can identify where GGB data can be found Can explain what community databases are and how they support agricultural research Can explain what model species are and why they are important for discoveries in agriculture Can talk about AgBioData and the main mission Can explain the challenges of GGB community databases and how they are addressing it Can explain how community databases promote equitable data in agricultural research	Ag UG, Grad

The curriculum primarily targets educators who teach UG and graduate students the basics of biological databases and FAIR principles. Throughout their careers, these students will search, download, analyse, and submit research data, work with databases, or curate data. Also targeted are scientists who wish to learn about the role of data repositories, how to use GGB resources, and how to implement FAIR guidelines when archiving and managing data in agricultural biological research. The curriculum, provided as Supplementary Data, is modular so that each unit can be viewed and used independently or sequentially. It begins at the UG level, with increasing rigor and detail added for graduate school/professional scientist audiences, and assumes basic knowledge of genetics, genomics, and breeding. All lessons are structured around a set of learning outcomes (Table 1; Supplementary Data) and include open-source slides and recordings of the lessons (https://zenodo.org/uploads/13641594). Some also include supporting materials, such as guided discussion points, exercise descriptions, and/or reflection topics to enable interested instructors to incorporate experiential learning and interactivity in the classroom. This diversity of learning modalities addresses the solicitation for various and complementary training materials on agricultural biological databases in the AgBioData qualitative survey mentioned above.

We envision the AgBioData Curriculum for FAIR Ag Data not as a static training material but rather as an evolving curriculum to which new lesson plans may be added following the rise of new data types or database-related topics (e.g. artificial intelligence) and suggestions from the AgBioData community.

### Biological databases and why we need them

Biological databases properly archive, manage, and provide access to the large amount of data constantly generated in biological research. They transform research data into reusable and valuable knowledge [17]. Just as traditional libraries have archived knowledge stored in text and numerical formats in professional journals, books, and similar publications, data libraries are fulfilling the same purpose by reviewing, curating, and making accessible data that otherwise would overwhelm traditional publications by their sheer size.

‘Biological database’ is one of the many overlapping terms (e.g. digital repository, database, web portal, website, resource, data repository, data bank, archive, and library). The nuances of these terms are explored in more detail in Lesson Plan 1 (Table 1), with consideration of their purpose (access vs. preservation), type of technology (data storage vs. data access vs. data analysis), and structure. For the remainder of this paper, we will use ‘biological database’ to refer to the collection of biological data and any interfaces that facilitate data search and access by scientists and computers by indexing, organizing, and optimizing the archived data [18]. GGB community databases are a subset of biological databases that handle genetic, genomic, and/or breeding data, and, as a particular focus for AgBioData, they are often used as examples in the curriculum.

Community databases rose in the 1990s and were initially organized around reference genome resources, explaining why some have ‘genome’ still reflected in their names. To spur the usefulness of these genomes, additional types of data have been integrated, such as gene functions, genetic markers, phenotypes, protein sequences, etc. This integration adds value to the individual data sets and improves their reach and impact on the research community. The earliest community databases include the Saccharomyces Genome Database (SGD [19]), the Arabidopsis Information Resource (TAIR [20]), Gramene [21], WormBase [22], MaizeGDB [23], the Mouse Genome Informatics (MGI [24]), and FlyBase [25].

New databases continue to be created with various scientific goals, and some have expired or are no longer maintained. To keep abreast of updates, scientists have several resources. The Nucleic Acid Research (NAR) journal publishes an annual issue of database articles with both new databases and updates to existing resources [26]. Re3data (https://www.re3data.org/), a registry of research data repositories, has 1861 records for life sciences, while FairSharing.org (https://fairsharing.org/) reports 1838 life science knowledgebases and repositories (last access September 2024). It is difficult to determine how many of these resources are still available and being updated. FairSharing reports that 1152 databases in its catalogue are ‘maintained’, while [27] nearly 70% of the NAR-published databases are active.

The database’s central objective often distinguishes recent database categorizations. For example, repositories may focus on long-term storage in contrast to knowledgebases that are curated and contain more explanatory information to facilitate discoveries. Community databases are often knowledgebases but also function as repositories that store long-term data. Some institutional and general data repositories span several categories. For instance, in 2015, the United States Department of Agriculture (USDA)—National Agricultural Library (NAL) created the Ag Data Commons, a generalist public repository for USDA-funded research data related to food and agriculture (https://data.nal.usda.gov/). Other examples of generalist repositories commonly used in biology are Figshare (https://knowledge.figshare.com/), Dryad (https://datadryad.org/stash), Zenodo (https://zenodo.org/), and Harvard Dataverse (https://dataverse.harvard.edu/), which all aim to make research data more findable, accessible, and citable. We explore all these aspects of biological databases in the educational curriculum to guide users in assessing which repository or repositories suit their needs (see Lesson Plans 1, 3, and 5 in Table 1).

### How do databases promote FAIR data?

**Figure 1.**
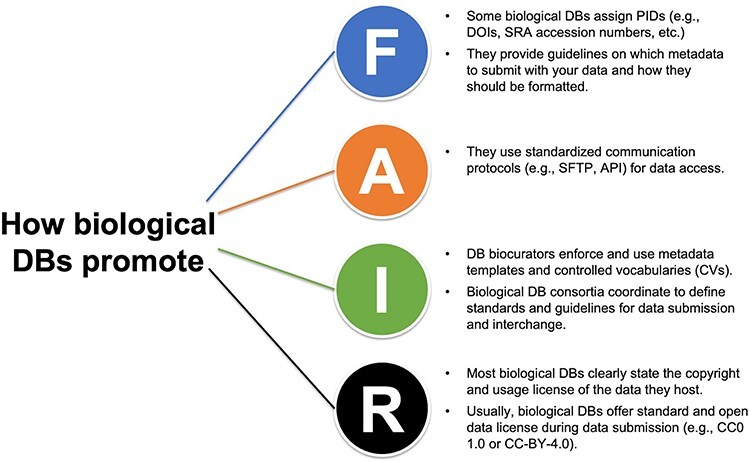
How biological databases (DBs) implement the FAIR principles and promote FAIR data.

The FAIR principles were introduced in 2016 by Wilkinson *et al*. [2] to address the common need to improve data discovery and reuse in the scientific community. There are 15 guiding principles, grouped into the categories of FAIR, which provide guidelines for research data and scholarly digital objects produced by humans and machines. By providing a space where data are archived, maintained, and made available for reuse, data repositories are essential for FAIR data (Fig. 1). We highlight this core concept in Lesson Plan 2 (Table 1), which overviews the FAIR principles with practical examples and exercises.

The first set of principles is designed to make the data more findable by human and machine-driven activities. This includes adopting unique and persistent identifiers to label the data and the related metadata. For instance, Ag Data Commons mints a digital object identifier (DOI) when a new dataset and its metadata are submitted, provided the data never received a DOI previously. Other examples of persistent identifiers used by biological databases are the accession numbers assigned to sequence datasets deposited at the NCBI Sequence Read Archive (SRA; https://www.ncbi.nlm.nih.gov/sra) or the proteome identifiers assigned by UniProt when a new assembly of a species and strain or subspecies is submitted (The UniProt Consortium, 2015 [28]).

The ‘findable’ FAIR principles also recommend submitting ‘rich’ metadata along with the data. Metadata are ‘data about the data,’ namely any type of information that describes the submitted data and facilitates its identification, understanding, and reuse. Most biological databases require or recommend the submission of metadata (e.g. sample location, ORCID of the researcher submitting the data, etc.). Recently, Deng *et al*. [29] reviewed the publicly funded repositories for raw and processed data used in plant genotype-to-phenotype studies and provided information on the metadata required during data submission by public crop community databases. Our curriculum also highlights the importance of applying guidelines and templates provided by biological databases for data submission, providing examples of good (and other) data sharing.

Even with a globally unique identifier and rich metadata, users may fail to find data relevant to their needs because they might not know where and how to look for the data. Our curriculum aims to help users identify and search in the appropriate databases and formulate search queries with proper terms In Lesson Plan 3, ‘Bio-Databases: Types of data, finding and obtaining data’ (Table 1), we provide guidelines and instructions on where to look for FAIR data and how to formulate search queries based on controlled vocabularies (CV) and ontologies, or any other standardized terms used in your field.

The second set of FAIR principles, data ‘accessibility’, focuses on how to access data and metadata through their persistent identifiers, emphasizing the importance of using standardized communication protocols for programmatic access. Our curriculum overviews the different protocols biological databases offer to retrieve the hosted data, including older use cases (e.g. Secure File Transfer Protocol; SFTP), the Application Programming Interface (API) system, and command-line utilities. By providing an overview of communication protocols implemented by databases to make data accessible, we aim to guide the users in choosing repositories that offer open-access data retrieval when sharing their data.

Another critical aspect of the FAIR principles is data ‘interoperability’, the ability to interchange and aggregate data from different resources meaningfully. Data interoperability is possible when data and metadata are formatted according to community-based standards, if available, and defined CVs and ontologies are implemented. Biocuration and the enforcement of metadata templates and CVs during data submission by biological databases promote and facilitate data interoperability. Biological repositories also coordinate defining standards and guidelines for data submission and interchange across platforms For instance, Tripal, an open-source toolkit for constructing online community databases [30], relies on CVs to facilitate data findability and access across all Tripal databases. Making data interoperable is a challenge for biology, where data is complex and difficult to model. Still, the benefits are potentially enormous, including new holistic, systems-level insights, and unprecedented scales of data integration.

It follows that when data can be easily found, accessed, and integrated, they are more easily reused. Data reuse is one of the ultimate goals of FAIR, and it is facilitated when data and metadata are richly described in a way that can be understood by the large public and provenance and usage licenses are clearly stated. This last set of FAIR principles, ‘data reuse’, encourages the inclusion of all types of attributes that can help a user (i.e. machine or human) understand the context under which the data were generated (e.g. How were the data processed? Where were they collected? etc.). Some database resources work to facilitate data reuse and integration: the Eukaryotic Pathogen, Vector and Host Informatics Resources (VEuPathDB.org; [31]; https://galaxyproject.org/) allows scientists to easily build workflows and run them on many datatypes found in archival repositories.

Licenses and other permissions are other critical features that significantly impact data reuse. Many biological repositories clearly state their host data’s copyright and usage license. For instance, NCBI ‘places no restrictions on the use or distribution of the data contained therein’ and does not accept ‘data that the submitter has requested restrictions on reuse or redistribution’, even if some submitters ‘may claim patent, copyright, or other intellectual property rights in all or a portion of the data (that has been submitted)’ (https://www.ncbi.nlm.nih.gov/home/about/policies/). Similarly, Dryad only accepts data that can be licensed under the Creative Commons Zero (CC0 1.0) waiver, which effectively puts the data into the public domain (https://datadryad.org/stash/best_practices). This is considered the best option for sharing data as it maximizes reuse potential by placing no restrictions upon it. We mention and review the most common license types in research in Lesson Plan 5, ‘Submitting data.’

**Figure 2.**
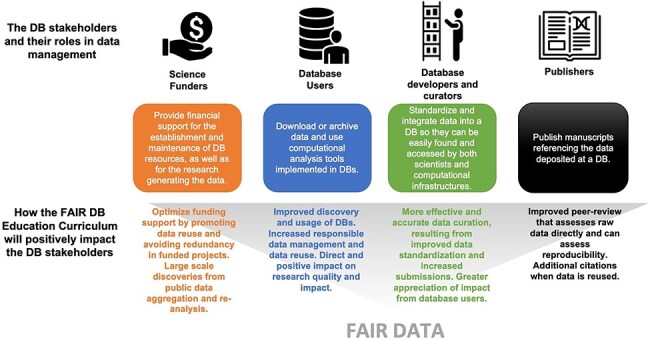
Database (DB) stakeholders, their roles in data management, and how the educational curriculum will positively impact them.

In summary, biological repositories make data more ‘findable’ by assigning digital identifiers and providing curated data and related metadata, ‘accessible’ by adopting standardized communication protocols, ‘interoperable’ by implementing community standards, and ‘reusable’ by integrating data with related resources and knowledge and defining their usage licenses (Fig. 2). In our curriculum, we cover all these aspects across several lesson plans, with the final aim of increasing current and future researchers’ awareness of how databases are essential components of FAIR data.

### How do biological databases support agricultural researchers?

As the amount of data has accumulated over more than a century in agricultural sciences, scientists, teachers, commercial and private organizations have recognized the need for systematic programs to gather, assess, curate, and make available these data. The increased rate of variability in climate and weather patterns, local and regional environmental conditions, food distribution systems and consumer concerns dictate even more generation and use of interconnected, large-scale understanding of food production systems. We have been using large databases in a number of ways for many years. We have developed computer systems which can store and analyse data in a variety of methodologies from relatively simple libraries of peer-reviewed scientific publications to complex data storage and analytical platforms in many different scientific disciplines. There is an essential need for an educated workforce and support system to develop, operate, and use our knowledge as encoded in these computer based systems.

In agriculture, the collation and use of data in dairy cattle breeding (The Dairy Herd Improvement Association, DHIA; https://dhia.org/) has been in use for more than a century to predict productive characteristics from ancestors’ performance [32, 33]. Many similar breeding databases in animal and plant agriculture are standard-setting and their use is standard practice. The National Research Council of the National Academies of Science, Engineering and Medicine (NASEM) has produced data compilations and application methods for over a century, and these are the scientific and legal standards for animal feeding requirements and human Recommended Dietary Allowance. It is now standard practice for scientists to check the latest summaries and recommendations of such database systems prior to conducting research, and it is often a requirement of funding agencies that such a consultation between the scientists and the database operators be conducted before setting the research hypotheses and applying for funding.

Ensuring the development and implementation of recommendations and standards of data sharing in agricultural research also falls within the mission of the AgBioData consortium, along with educating the research community on the efforts behind and the use of GGB database resources. Many databases in agricultural research are community-specific resources (Supplementary **Table S1**) that focus on a single species or taxonomically related species, such as GrainGenes, a database for small grains [34], or the Pulse Crop Database (PCD), serving the research community working with pulse crops (https://www.pulsedb.org/). Some community databases focus on specific classes of data, such as metabolism (e.g. the Plant Metabolic Pathway Database [35], ontologies (e.g. Planteome [36]), and germplasms (e.g. the USDA-ARS Germplasm Resources Information Network—GRIN [37]). Thanks to these resources, many discoveries directly impacting agriculture are now possible, ranging from genome comparisons across closely related species to the molecular screening for genetic resistance in a crop germplasm collection to gene cloning and editing.

Agricultural biological databases provide fundamental knowledge not only to scientists but also to breeders, publishers, and all the stakeholders involved in agricultural research. They connect laboratories, foster collaborations, and minimize the duplication of research efforts. We hope that the modular curriculum presented here will help scientists and students understand and support database use in all aspects of improving our global food system.

### How the AgBioData curriculum for FAIR Ag Data will impact the biological database stakeholders

Database resources serve different stakeholders, including database users, database staff (e.g. curators and developers), database funders, and scholarly publishers (Fig. 1). A ‘database user’ is anyone who accesses the biological database for downloading, searching, viewing, submitting, or archiving data, as well as running any other computational analysis tool implemented in the database. We aim to increase database users’ awareness of the importance of FAIR data and educate them to find, manage, and share their data responsibly so they are findable and accessible to anyone.

Another key figure in the database ecosystem is the ‘database curator’ or ‘biocurator’. Biocuration generally involves reading peer-reviewed scientific articles, extracting potentially valuable datasets, and integrating these data into data repositories in a way that they can be easily found and accessed by both scientists and computational infrastructures [1]. Biocuration is necessary to standardize data and make it computationally accessible. For example, a biocurator might improve data accessibility by assigning standard ontology terms to data or metadata, or they might make a data set more interoperable by converting it to a standard format. Biocurators may be employed by the database or may be members of the research community to maintain and enhance data coverage and accuracy in a database [38].

While biocuration is a particular profession, anyone who does data cleaning, organization, standardization, and integration performs biological data curation. Much of modern biology is computationally intensive, often requiring accessing and integrating massive ‘omics’ datasets. Therefore, researchers need to develop bioinformatics skills, including proper data management and curation to manage their work effectively. One of the goals of this curriculum is to teach researchers principles of FAIR data management. Not only it will aid in their own research, but it will also facilitate and smooth the work of professional biocurators aiming to integrate research products into curated databases. Despite the establishment of a professional society of biocuration (the International Society for Biocuration—ISB; https://www.biocuration.org/), which offers visibility, training materials, and career opportunities, the role of biocurators, and their crucial efforts for research success are still unknown to many database users. Another goal of the included curriculum is to teach biocuration as a cornerstone of research in life science to database users [39].

Other key stakeholders of biological databases targeted by our curriculum are ‘database funders’ and scholarly ‘publishers’. ‘Database funders’ provide financial support for the establishment and long-term maintenance of biological databases and can be public (e.g. governmental agencies) or private institutions. Often, GGB databases lack long-term financial support, which can cause permanent loss of valuable data and software gathered and built at taxpayer or industry expense, slowing the progress of research. Universities, independent research organizations, lab groups, and even individuals may be able to extend the life of a database through subscriptions, but this is a relatively new funding model for GGB databases that might not be ideal for all agricultural research communities. A GGB database access granted only to subscribers might exclude less wealthy research institutions and communities. The AgBioData consortium and other initiatives (e.g. the Global Biodata Coalition; https://globalbiodata.org/ [40]) are working on this critical issue to ensure data persistency and longevity. Database funders also pay for research that generates the data submitted to GGB databases. Guidelines and best practices of research data management can optimize funding support by promoting data reuse and, therefore, avoiding redundancy in the funded projects.

‘Scholarly publishers’ and the reviewers and editors who are part of that workflow are essential elements in assessing data quality and can play a critical role in supporting FAIR data. They determine which data are accepted as new knowledge, often encourage data standards, and require the submission of peer-accepted data into particular databases. The usefulness of databases depends upon a robust peer review system to determine the curation and use of data. Non-peer-reviewed data can be and often should be included in databases, but only when clearly identified as such.

‘Biological repositories’ may be mutual stakeholders because they reuse each other’s data or work together on a specific mission, such as developing community-based standards of data sharing or establishing a database consortium. Examples are the INSDC [41], the Alliance for Genome Resources (AGR [42]), and AgBioData [3]. Finally, it is worth mentioning the ‘general public’ as a further stakeholder of the database ecosystem; access to curated and high-quality data are needed for the development of public-facing dashboards that allows the public to easily interact with the data, as we have seen during the COVID-19 pandemics [43]. Specifically for agricultural research, the ‘general public’ includes farmers and food consumers, which are more and more interested in staying informed on the most recent discoveries and their impact.

## Conclusions and future directions

The AgBioData Curriculum for FAIR Ag Data addresses the need for educational material on biological databases and their use for FAIR data management in agricultural research. The seven lesson plans cover different applications of biological database resources that database users can perform, starting with the proper choice of a biological database resource, to how to efficiently find, download, and submit data, to an introduction to DMP and GGB community databases, all according to the FAIR principles. This curriculum highlights the crucial role of GGB community databases in agricultural research, aiming to increase scientists’ awareness of how much of their work relies on these resources.

We hope that the AgBioData curriculum for FAIR Ag Data and the mentors who will decide to use it in their classroom will foster, recognize, and support interest in computational biology and bioinformatics at all levels. With the curriculum’s lesson plans, we encourage class discussions on how the FAIR principles intersect with justice, equity, diversity, and inclusion issues. Biological databases address global inequity as technological resources that are usually freely available to biologists worldwide. Many students from low-income countries do not have adequate computational resources, but with high-speed internet and databases that offer open access data and analysis tools that run on remote servers and/or the cloud, these scientists may still conduct bioinformatic research. Thus, biological databases can break technological and financial barriers to research while connecting researchers and students across the globe and promoting new groundbreaking discoveries through data reuse [44].

The overall goal of the curriculum is to foster a new generation of data scientists who understand and promote FAIR data and use, support, and build databases that are essential to the FAIR principles. Lessons are posted on the AgBioData Zenodo community (https://zenodo.org/records/14278084) and are freely available without restriction in formats that are accessible and adaptable. We encourage using these materials in whatever way suits the community, from use in UG and graduate classrooms to self-guided discovery.

We have already presented the curriculum at national and international conferences (e.g. the Plant and Animal Genome Conference and the Annual Plant Biology Conference). We will continue to disseminate the modules and promote adoption broadly. We invite the AgBioData community and anyone interested to incorporate the lesson plans into existing or new UG and graduate courses and supply any modifications and assessments that will be used to improve the curriculum. Also, in the next 2 years, we will initiate a championship programme to recruit and support faculty and students who wish to either incorporate the modules in their courses or use them as self-guided lessons for lab members. Through this program, the annual meetings, and conference workshops, we will get feedback on the lessons and improve and update the curriculum on new subjects emerging in the GGB database ecosystem. The EWG, the AgBioData Steering Committee, and the project coordinator will continue leading the curriculum development and improvement and establishing collaboration with other initiatives to translate and expand the learning modules.

## Supplementary Material

baaf034_Supp

## Data Availability

Lessons are posted on the AgBioData Zenodo community (https://zenodo.org/records/14278084) and are freely available without restriction.
