# Supplementary material for: A teaching and training framework to promote findable, accessible, interoperable, and reusable data generation in agriculture
Source: Database (Oxford). 2025 Apr 25;2025:baaf034. doi: 10.1093/database/baaf034 (PMC12024112; doi:10.1093/database/baaf034)
Supplement: baaf034_Supp [file baaf034_supp.zip › suppl_data/Supplementary_Data_Marrano_et_al_AGB_FAIR_Ag_Curriculum.docx]

Supplementary Data of “A teaching and training framework to promote FAIR data generation in agriculture”

AUTHORS:

Annarita Marrano^1^ http://orcid.org/0000-0001-9560-2706

Leyla Cabugos^2^ https://orcid.org/0000-0003-4031-3987

Alenka Hafner^3^ https://orcid.org/0000-0003-4262-9176

Beant Kapoor^4^ https://orcid.org/0000-0002-0762-4639

John McNamara^5^ https://orcid.org/0000-0003-0021-2006

Megan O'Donnell^6^ https://orcid.org/0000-0002-4632-6642

Leonore Reiser^1^ https://orcid.org/0000-0003-0073-0858

Marcela Karey Tello-Ruiz^7^ https://orcid.org/0000-0002-7499-5368

Huiting Zhang^5^ https://orcid.org/0000-0003-1741-9492

Meg Staton^4^ https://orcid.org/0000-0003-2971-9353

^1^ Phoenix Bioinformatics, Newark, CA

^2^ California Polytechnic State University, San Luis Obispo, CA

^3^ Pennsylvania State University, State College, PA

^4^ University of Tennessee, Knoxville, TN

^5^ Washington State University, Pullman, WA

^6^ Iowa State University, Ames, IA

^7^ Cold Spring Harbor Laboratory, Cold Spring Harbor, NY

## Lesson Plan 1: What is a biological digital repository?

**Lead:** Meg Staton

**Primary audience(s):** HS, UG, Masters, PhD

### Learning outcomes:

- Can explain the terms digital repository, database, web portal, website, resource, data repository, data bank, archive, library and their overlap
- Can explain why databases were originally developed and how they support the research community today
- Can explain the spectrum of scientific databases from large, general purpose (Dryad, figshare) to targeted, community-specific (TAIR, other AgBioData repos)
- Can explain and discuss the relationships among the terms open data, primary data, secondary data, metadata, data provenance, data management, data curation
- Can explain the relationship between data producers, data users, open data, and technology

### Summary of Tasks/Actions:

- Many terms are used to describe information resources, including digital repository, database, web portal, web site, resource, data repository, data bank, archive, library and their overlap.
  - Information resource is general enough to be used as an umbrella term for all of these but also for much, much more. More specificity is likely beneficial.
  - These terms are often used interchangeably and differences are subtle. Further, the terms may have somewhat different common usages depending on the discipline. For example, “database” from the computer science discipline is used to refer to software that stores information on a computer and is concerned with data representation, storage structure, and basic querying language, largely divorced from user-centric access. In agriculture, “database” is a more all encompassing term referring to all of the technology and access methods for an entire resource or website, i.e. TAIR is a database. In another example of discipline-specific definitions, information scientists may strictly differentiate library vs archive (<https://casrai-test.evision.ca/rdm-glossary/>). Generally, end users apply terms more loosely and with a more all encompassing meaning than information and computer domain scientists.
  - Further faceting principles for these terms:
    - What is the purpose of the resource? Emphasis on access vs long term preservation (repository vs archive)
    - Is the term referring to the data itself, the method (user interface) to access the data, or both? Database vs web site
    - What is the method of access? Physical (centralized), physical (local), internet (computer/mobile/IoT), stand alone software (older model). Library vs archive vs web portal vs app.
    - Repository (access/persistence, structure desirable but optional, not necessarily just data, think for example software in github, searching for a whole dataset) vs database (general structured data, could be as simple as excel, integrating across datasets, looking for specific content)
- Databases were originally developed to support large datasets and data sharing. They continue to be important to research, and are growing in scope due to high throughput data generation and high performance computing.
  - 1987-1988 - NCBI/DDBJ/ENA
  - 1990s - FlyBase, TAiR, treegenes, etc
  - Early 2000s - GMOD
  - Cooray 2012
  - Protein data bank - Bourne 2003
  - Longevity issue - Attwood 2015
  - Leonelli - Model Org DBs
- Scientific databases each have a unique scope, target audience, and data management strategy. This ranges from large, general purpose databases (Dryad, figshare) to targeted, community-specific (TAIR, other AgBioData repos).
- Understands the difference between preservation repository, access repository, and catalog record *(make sure our terminology is consistent)*
  - Catalog: will point to data that lives elsewhere - either another server or repository within the same organization or to a completely different organization
  - Repository: Holds the data directly
  - Preservation: Holds the data directly but will include long-term plans, information, contingencies, and specify how long data will be kept. These repositories must meet specific criteria for safe long-term storage. i.e. CoreTrustSeal certification, ISO 16363 certification, TRAC certification, etc.

Suggested activities:

- Explore data related to a single organism in different databases. For example, Prunus persica (peach) in FigShare, NCBI, and Genome Database for Rosaceae. Discuss what specific informational resource term would fit each database, who the target audience of the database is, and the types of data stored in each.
- Examine several sites and determine which category they fall (preservation, access, catalog record) - be able to articulate WHY you came to those conclusions

### References:

- Cochrane G, Karsch-Mizrachi I, Takagi T, Sequence Database Collaboration IN. The international nucleotide sequence database collaboration. Nucleic acids research. 2016 Jan 4;44(D1):D48-50.
- Baxevanis AD, Bateman A. The importance of biological databases in biological discovery. Current protocols in bioinformatics. 2015 Jun;50(1):1-.
- Caswell J, Gans JD, Generous N, Hudson CM, Merkley E, Johnson C, Oehmen C, Omberg K, Purvine E, Taylor K, Ting CL. Defending our public biological databases as a global critical infrastructure. Frontiers in bioengineering and biotechnology. 2019 Apr 5;7:58.
- Cooray MP. Molecular biological databases: evolutionary history, data modeling, implementation and ethical background. Sri Lanka Journal of Bio-Medical Informatics. 2012 Oct 31;3(1).
- Leonelli S, Ankeny RA. Re-thinking organisms: The impact of databases on model organism biology. Studies in History and Philosophy of Science Part C: Studies in History and Philosophy of Biological and Biomedical Sciences. 2012 Mar 1;43(1):29-36.
- Sharma PK, Yadav IS. Biological databases and their application. InBioinformatics 2022 Jan 1 (pp. 17-31). Academic Press.
- Smith TF. The history of the genetic sequence databases. Genomics. 1990 Apr 1;6(4):701-7.
- <https://www.coretrustseal.org/>
- <http://www.iso16363.org/standards/iso-16363/>
- <https://www.crl.edu/sites/default/files/d6/attachments/pages/trac_0.pdf>
- <https://www.hathitrust.org/trac>

## Lesson Plan 2: FAIR and databases

**Lead:** Annarita Marrano

**Primary audience(s):** HS, UG, Masters, PhD

### Learning outcomes:

- Can paraphrase the acronym FAIR.
- Can explain why the FAIR principles are important and when they have been introduced.
- Can give a general overview of the FAIR principles, explaining what digital objects they apply to, their history, and why they have been introduced.
- Can describe the guidelines for making a digital object Findable.
- Can explain what a persistent identifier is and why it is important.
- Can define the guidelines related to the “Accessible” principles.
- Can explain when an object is interoperable and what are the most common Conserved Vocabularies used by biological databases (e.g., Gene Ontology, Trait Ontology, etc.)
- Can explain how biological databases relate to FAIR guidelines.
- Can paraphrase the CARE principles, describe them, and why they are complementary to the FAIR principles.

### Summary of Tasks/Actions:

- Introduction to FAIR principles
  - Wilkinson et al. 2016 introduce the *FAIR Guiding Principles*, a list of 15 guidelines elaborated around four core principles: data Findability, Accessibility, Interoperability, and Reusability.
  - These principles apply to every type of scholarly digital objects archived in a repository (e.g., data, metadata, algorithm, software, protocol, etc.).
  - The FAIR principles facilitate both human-driven and machine-driven activities. In particular, most of today's research is automated or relies on computational agents that need to recognize and sort a wide range of data formats and information. The FAIR principles make this enormous process of data sorting and indexing simpler, more rapid, and more accurate.
- Findable
  - Your data are well described and can be found by anybody in a searchable resource.
  - What is a globally unique and persistent identifier?
    - DOI - digital object identifier
    - ORCID

Suggested activities:

- - - identify good and bad examples of DOIs;
    - match DOI with the right document;
  - What are the metadata?
    Suggested activities:
    - based on the primary audience, select a dataset type (e.g., marker panel) and ask what type of descriptors are necessary for the data to be findable (e.g., genome location; allele; genotypes; accession numbers; reference genome used to identify the markers; etc.)
    - Try to upload a dataset in one database and see which type of metadata it asks to upload
- Accessible
  - This principle focuses on how the data and metadata are retrievable using their persistent identifiers.
  - Accessible does not mean ‘open’, but implies that you provide clear and simple conditions for both humans and machine to access your data.

Suggested activities:

- - - Try to download data from a repository using SFTP protocol
- Interoperable
  - Human and computer system should be able to interpret and exchange your data and its associated metadata.
  - It is recommended that, when formatting your (meta)data, you follow standardized protocols and use controlled vocabularies understandable from a large community.
  - Examples of standardized format and controlled vocabularies:
    - [IUPAC nomenclature code for nucleotides and amino acids](https://www.bioinformatics.org/sms/iupac.html)
    - [Gene Ontology](http://geneontology.org/docs/ontology-documentation/)
    - [Plant Ontology](https://planteome.org/)

Suggested activities:

- - - provide different formats of genotypic files (*e.g.*, numeric, AB, nucleotide) and debate which one can be interpreted by a large community.
    - Practice with the Plant Ontology portal
- [Reusable](https://www.go-fair.org/fair-principles/r1-metadata-richly-described-plurality-accurate-relevant-attributes/)
  - In order to make your data actually reusable by other scientists, you need to provide any type of attributes that can inform a user (machine or human) about the context under which your data were generated (*e.g.*, provenance, protocols, usage license, program or machines used, species, etc.).
  - Format the data and metadata according to your community’s standards, if they are available. Examples:
    - [MIAPE](https://en.wikipedia.org/wiki/Proteomics_Standards_Initiative) (Minimal Information About Proteomics Experiments)
    - [NCBI Prokaryotic Genome Annotation Standards](https://www.ncbi.nlm.nih.gov/genome/annotation_prok/standards/)

Suggested activities:

- - - A list of attributes to match with putative submitted data (*e.g.*, what type of attributes should we provide when publishing a GWAS study).
    - Watch the “Data sharing and management Snofu in 3 short act” video (<https://youtu.be/N2zK3sAtr-4>).
- Just as the FAIR principles guide data users and generators towards good data management and stewardship, the CARE (Collective benefits, Authority to control, Responsability, Ethics) principles encourage to include the ‘people’ from and for which the data are taken, in the actions and discussion about data access and management. The CARE principles have been introduced by the International Data Sovereignty Interest group of the Research Data Alliance (RDA) and are “people and purpose-oriented, reflecting the crucial role of data in advancing innovation, governance, and self-determination among Indigenous People” (Carroll et al. 2020).
- When we talk about Indigenous data, we refer to:
  - Data about Indigenous non-human relations (e.g., land, plant, animals, ecosystems, etc.)
  - Data about Indigenous people as individuals (e.g., health, corporate, etc.)
  - Data about Indigenous traditions, culture, and information
- The CARE principles include a total of 12 guidelines that can be divided into four categories (from the “Global Indigenous Data Alliance. (2022). ‘Indigenous Data Sovereignty and Governance.’ Carroll, Cummins, and Martinez):
  - ***Collective Benefit:*** Data ecosystems shall be designed and function in ways that enable Indigenous Peoples to derive benefit from the data.
  - ***Authority to Control:*** Indigenous Peoples’ rights and interests in Indigenous data must be

recognised, and their authority to control such data should be empowered. Indigenous data

governance enables Indigenous Peoples and governing bodies to determine how

Indigenous Peoples, as well as Indigenous lands, territories, resources, knowledge and

geographical indicators are represented and identified within data.

- - ***Responsibility:*** Those working with Indigenous data have a responsibility to share how those data are used to support Indigenous Peoples’ self-determination and collective benefit. Accountability requires meaningful and openly available evidence of these efforts and the benefits accruing to Indigenous Peoples.
  - ***Ethics:*** Indigenous Peoples’ rights and well-being should be the primary concern at all stages of the data life cycle and across the data ecosystem.

### References:

1. Wilkinson MD, Dumontier M, Aalbersberg IJ, Appleton G, Axton M, Baak A, Blomberg N, Boiten JW, da Silva Santos LB, Bourne PE, Bouwman J. (2016) The FAIR Guiding Principles for scientific data management and stewardship. *Scientific data*, 3(1):1-9.
2. Sprague, E.R.. Orcid. (2017) *Journal of the Medical Library Association: JMLA*, 105(2):207.
3. Cooper, L., Walls, R.L., Elser, J., Gandolfo MA, Stevenson DW, Smith B, Preece J, Athreya B, Mungall CJ, Rensing S, Hiss M. The plant ontology as a tool for comparative plant anatomy and genomic analyses. (2013) *Plant and Cell Physiology*. 54(2):e1-.
4. Taylor CF, Paton NW, Lilley KS, Binz PA, Julian Jr RK, Jones AR, Zhu W, Apweiler R, Aebersold R, Deutsch EW, Dunn MJ. The minimum information about a proteomics experiment (MIAPE). (2007) *Nature biotechnology*, 25(8):887-93.
5. Chun J, Oren A, Ventosa A, Christensen H, Arahal DR, da Costa MS, Rooney AP, Yi H, Xu XW, De Meyer S, Trujillo ME. Proposed minimal standards for the use of genome data for the taxonomy of prokaryotes. (2018) *International journal of systematic and evolutionary microbiology*, 68(1):461-6.
6. Saha S, Cain S, Cannon EK, Dunn N, Farmer A, Hu ZL, Maslen G, Moxon S, Mungall CJ, Nelson R, Poelchau MF. Recommendations for extending the GFF3 specification for improved interoperability of genomic data. (2022) *arXiv*, 2202.07782.
7. Carroll S, Garba I, Figueroa-Rodríguez O, Holbrook J, Lovett R, Materechera S, Parsons M, Raseroka K, Rodriguez-Lonebear D, Rowe R, Sara R. The CARE principles for indigenous data governance. (2020) *Data science journal*, 19.

## Lesson Plan 3: Bio-Databases: Types of data, Finding, and Obtaining data

**Lead:** Beant Kapoor, Alenka Hafner, Annarita Marrano

**Primary audience(s):** HS, UG, Masters, PhD

### Learning outcomes:

- Understand the diversity of **data types** archived and managed in biological databases
- Learn what **biocuration** is and why it important
- Learn how and where to **find data**
  - Defining a research question that can be answered by searching biological databases
  - Finding resources that meet the needs of the research question (e.g., [re3data,](https://www.re3data.org/) [FAIRsharing](https://fairsharing.org/))
- Learn how to **obtain data** using Graphical User Interface (GUI) and Command Line Interface (CLI) tools

### Summary of Tasks/Actions:

1. **Data types:**
   - Experimental data are generated after planning and performing a controlled scientific experiment. In biology, there is a large diversity of experimental data types generated, ranging from sequence data (e.g., DNA, RNA), to protein structures to phenotypic measurements to literature. These data are managed and curated at different biological database resources. For instance, DNA databases centre on managing DNA data from many or specific species. Examples of DNA databases are GenBank, DDBJ, and ENA. [The Universal Protein Resource (UniProt)](https://www.uniprot.org) is a comprehensive resource for protein sequence and annotation data. The UniProt databases are the [UniProt Knowledgebase (UniProtKB)](https://www.uniprot.org/help/uniprotkb), the [UniProt Reference Clusters (UniRef)](https://www.uniprot.org/help/uniref), and the [UniProt Archive (UniParc)](https://www.uniprot.org/help/uniparc) (<https://doi.org/10.1093/nar/gku989>). Other examples of protein databases are the [EMBL-EBI InterPro](https://www.ebi.ac.uk/interpro/), which provides functional analysis of proteins by classifying them into families and predicting domains and important sites, and the [RCSB Protein Data Bank (RCSB PDB)](https://www.rcsb.org). The [KEGG PATHWAY](https://www.genome.jp/kegg/pathway.html) database collects manually drawn pathway maps representing our knowledge of the molecular interaction, reaction and relation networks (<https://doi.org/10.1002/0470857897.ch8>).
2. **Biocuration**: Most biological databases curate their data. “*Biocuration involves the translation and integration of information relevant to biology into a database or resource that enables integration of the scientific literature as well as large data sets*” (from the International Society for Biocuration; <https://www.biocuration.org/>). The process of biocuration might change across databases but generally involves i) the collection of scientific data, ii) quality-check and validation of the information collected, iii) organizing the data in a logical, consistent and relevant manner and iv) knowledge integration into databases. The professionals behind this detailed work are the biocurators, whose efforts to extract, interpret, summarize, and archive data from a variety of sources guarantee the integrity of data, helping to create knowledge. Thanks to biocuration, data are more discoverable, interoperable, and accessible from the research community.

Suggested activities:

- - - Watch the biocuration triage of The Arabidopsis Information Resource (TAIR)

1. **Finding data:**

First steps to finding information on biological systems:

- - The questions that one needs to answer before beginning the search for biological data are:
    - What is the research question?
    - Do we expect/want the answer to focus on just one taxon/organism/gene/organism/etc.?
    - What is the most appropriate (combination) of search terms?
      1. The importance of controlled vocabularies and ontology
      2. What type of ontologies are available for biological data
    - Where to look for the data (primary, secondary, composite, or organism-specific database)
    - What type(s) of data do we expect to appear in the answer (e.g. sequencing run, summary page for a gene, papers on the topic)?
    - How to define a good research question
    - How to find a resource that will meet your needs
      1. Whole internet search vs targeted resource search (vs literature search?)

*Gregory et. al (2018)* highlight the importance of thinking about the exact characteristics of the data you require so that you save time as the scope of your search is narrowed. If your research question is restricted to one species, especially in the case of a model one, looking for an organism-specific database will likely yield the best results. Similarly, domain-specific portals offer a collection of data resources that facilitate broad searches within a discipline. In the agricultural field, this is exemplified by the AgBioData consortium, which involves more than 40 GGB database resources (<https://www.agbiodata.org/databases>).

Another example is the REgistry of REsearch data REpositories (<https://www.re3data.org/>), which allow you to find the right database where to submit or find your data, and FAIRsharing (<https://fairsharing.org/>), a registry of FAIR knowledgebases and repositories of data and other digital assets. A broad search of a different scope may target all genes and proteins of a family (regardless of taxonomy) or all data deposited to a public repository from a published paper. For these searches, databases and repositories listed on NIH’s National Library of Medicine - National Center of Biotechnology Information (<https://www.ncbi.nlm.nih.gov/guide/all/>) are likely to provide the needed data collection.

Once you have identified one or more databases as tools in your search, it is advisable to familiarize yourself with its scope, organization, curation and search functions. This will aid in constructing the most helpful search query as well as ensure that you are aware of the limits and limitations of the results you can expect.

1. Think about your expectations of results - paper, dataset, specific type of information. Important in order to evaluate results
2. Evaluate quality of data
3. Keyword search vs nucleotide vs protein, Search query algorithms

Database search tips:

- - Boolean operators serve as the foundation for mathematical sets and database logic. They combine your search terms to narrow or broaden your results list. The three basic boolean operators are **AND**, **OR**, and **NOT**.
    - **AND**: narrows the results and instructs the database that **ALL** search phrases must appear in the displayed results. For example -
      1. plant **AND** microbiome **AND** 2022 - will display only those results which contain all of these search phrases
    - **OR**: broadens the results and tells the database that **at least ONE** search term must be included in the results. For example -
      1. bud break **OR** marcescence - will display all those results which contain either of these search terms or both.
    - **NOT**: narrows the results and tells the database to ignore concepts that search terms might imply. For example -
      1. structural variation **NOT** human - will return results that include “structural variation” but excludes “human”

Suggested activities:

- **HS & UG:** Your query term is “sonic hedgehog”. Compare search results from Google and your chosen database. These could include, for example, NCBI gene, NCBI protein, PubMed or an organism-specific database, such FlyBase, TAIR etc. A class discussion about the limitations of internet search engines when retrieving specialized knowledge and the benefits of biological databases should follow. Dissecting the results from specialized databases allows revision of material from the previous lesson and their practical application.
- **UG:** A practical run through of all the steps necessary to find biological data that will answer a specific question:
  1. Define a short research question (a useful demonstration is to have more than one student try to answer the same question) - e.g. What are the known interactor of the human Sonic Hedgehog gene (SHH)?
  2. Discussion about what students can expect to learn from a literature database vs. a biological data database and what type of data they expect will answer their question.
  3. Everyone creates a step-wise description of their search (i.e., which databases they searched first, what did they find, useful hyperlinks to other databases etc.) and a clear answer to their research question (e.g. a list of SHH)
  4. A class discussion comparing the results, answering the same question and creating a master list of useful databases the students found in their search.
- **GRAD (Masters & PhD):**
  1. Decision trees on defining the query search, identifying the right resource, and obtaining the data.
  2. Accessing the raw data of a sequencing run from a recent paper and running quality checks appropriate for that dataset.
     1. Example 1: RNA-seq data retrieval from Sequence Read Archive or Gene Expression Omnibus. A great protocol for retrieval and assessing quality with the common tool FastQC was written by Chatterjee et al. (2018)
  3. Suggested paper to read (<https://www.nature.com/articles/s41467-022-29584-y>) and find out -
     - 1. What types of data were generated?
       2. Where was the raw data submitted?
       3. Where was the genome submitted? Were you able to find the location easily? Can you download the genome in the format you want?
       4. Is there enough metadata provided to understand the generated product?

1. **Obtaining Data:**

- There are different systems that you can use when downloading data from a biological digital repository, and most of them can be divided in two main categories:
  - Graphical User Interface (GUI) tools
  - Command Line Interface (CLI) tools.
- GUI tools allow you to interact with the database through icons, menus, and data. It is easier for a user navigate the database and it does not require skills in programming languages. To download your data, you simply click on a File Transport Protocol (FTP) or HTTP link.
  - FTP is a standard communication protocol used fortransferring data from a server to a client on a computer network. HTTP is the foundation of data communication on the web; the user can easily access data by mouse clicking hyperlinks to other resources where the data are physically stored.
  - GUI tools are user-friendly, since they don’t require the user to have programming skills. The user can easily navigate the database and find the data thanks to the visual display. However, GUI-based databases can be slower in the data download than a CLI-based interface, and need a relatively high among of data storage space in the system.
  - An example of GUI tool is Globus (<https://www.globus.org/>), a comprehensive toolkit for building and managing large datasets. It provides essential tools for secure authentication, job management, data transfer, and resource discovery across distributed computing environments. The AgBioData Data Federation Training working group developed training material on Globus, available at <https://github.com/AgBioData/DataFederation_WG/wiki/Globus>.
- CLIs are interfaces you use to interact with your operating system through your keyboards. Every operating system has a CLI; for instance, macOS has the Terminal application, while Windows uses PowerShell. Popular commands you can use are, for instance, ‘pwd’ to see in which directory you are, ‘ls’ to list all the files and folders present in the directory you are in, and ‘cd’ to change directory. For a deeper knowledge of command line interfaces, we recommend to attend specialized classes at your institution or online workshops (e.g., the free Data Carpentry workshop“Introduction to the Command Line for Genomics”; <https://datacarpentry.org/shell-genomics/>).
- CLIs are usually faster and more efficient than GUI tools since they require less memory. They provide remote access to servers and every step of the process is documented. However, before using a CLI, you need to train on command line languages and this can take some time. Also, when using CLI you need to be accurate to avoid overwriting files or getting the wrong output.
- Therefore, for downloading data using a CLI on your laptop, you will need to type commands in a shell, which is an intermediary between you, the operating system of your laptop, and the remote database. A command used for downloading data from a database is ‘wget’, which stands for ‘world wide web and get’.

Suggested activities:

- Go to NCBI (<https://www.ncbi.nlm.nih.gov/>) and select ‘Genome’ in the drop-down menu on the left of the search bar. Look for the Arabidopsis thaliana tiar10 genome. Right click on the genome assembly named ‘TAIR10.1’ and select ‘Copy link”. Then open a *shell* window, type ‘wget’, and paste the link. Press ‘Enter’ and wait for the download to finish. In which format was downloaded your file? What do you see when you open it? Are you familiar with a FASTA file?

### References

1. Benson DA, Cavanaugh M, Clark K, Karsch-Mizrachi I, Lipman DJ, Ostell J, Sayers EW. GenBank. (2012) *Nucleic acids research*, 41(D1):D36-42.
2. Oshlack A, Robinson MD, Young MD. (2010) From RNA-seq reads to differential expression results. *Genome Biology*, 1-0.
3. Han Y, Gao S, Muegge K, Zhang W, Zhou B. Advanced applications of RNA sequencing and challenges. (2015) *Bioinformatics and biology insights*, BBI-S28991.
4. Zou D, Ma L, Yu J, Zhang Z. Biological databases for human research. (2015) *Genomics, proteomics and bioinformatics*, 13(1):55-63.
5. Brazma A, Hingamp P, Quackenbush J, Sherlock G, Spellman P, Stoeckert C, Aach J, Ansorge W, Ball CA, Causton HC, Gaasterland T. Minimum information about a microarray experiment (MIAME)—toward standards for microarray data. (2001) *Nature genetics*, 29(4):365-71.
6. Rustici G, Williams E, Barzine M, Brazma A, Bumgarner R, Chierici M, Furlanello C, Greger L, Jurman G, Miller M, Ouellette BF. Transcriptomics data availability and reusability in the transition from microarray to next-generation sequencing. (2021) *bioRxiv*, 2020-12.
7. Holinski A, Burke ML, Morgan SL, McQuilton P, Palagi PM. Biocuration-mapping resources and needs. (2020) *F1000Research*, 9.
8. Gregory K, Khalsa SJ, Michener WK, Psomopoulos FE, De Waard A, Wu M. Eleven quick tips for finding research data. (2018) *PLoS Computational Biology*, 14(4):e1006038.
9. Chatterjee A, Ahn A, Rodger EJ, Stockwell PA, Eccles MR. A guide for designing and analyzing RNA-Seq data. (2018) *Gene expression analysis: methods and protocols*, 35-80.

## Lesson Plan 4: Creating and sharing trustworthy data

**Lead:** Megan O’Donnell

**Primary audience(s):** UG, Masters, PhD

### Learning Outcomes:

After completion of this unit, students will be able to:

- Summarize how trust is a core part of data and database appraisal.
- Articulate how good data management assures good, and trustworthy, data sharing.

### Summary of Tasks/actions

1. TRUST
   1. The TRUST (Transparency, Responsibility, User focus, Sustainability, Technology) portion of the lesson is centered on information source appraisal - i.e., critically examining where information came from and if it meets your quality expectations. Instructors are encouraged to use the questions [on the slides](https://docs.google.com/presentation/d/1q-i2cO6QmFm0kAP6sfWOrCyHTOVQd7krti-Se5HOC6w/edit?usp=sharing) and in the suggested tasks section for class discussion. During discussions make sure to emphasize the importance of being able to find information about data sets through metadata and in attached files such as readmes, codebooks, and data dictionaries. Personal examples from your own experience can reinforce the importance and value of this information.
2. Data management and sharing plans
   1. The data management and sharing plan section brings together FAIR, TRUST, data management plans (DMPs) and (hopefully) the students’ own experience attempting to locate and/or reuse someone else’s data.
   2. Upper-division students who are collecting and managing their own data, or using data for a project, would do well to try to write a data management plan while lower-division students should focus on the what and why of the plans.
3. DMPs and TRUST together form a system that asks researchers to produce and share well-organized, documented, and transparent, data. To do this, they need trustworthy systems.
   1. While good data management can be achieved without TRUST, trustworthy scientific data sharing cannot.
   2. Compare and contrast how [Kaggle presents and documents](https://www.kaggle.com/datasets/nidzsharma/covid-19-variant-data) a public COVID-19 data set compared to how Harvard’s Dataverse [does the same](https://doi.org/10.7910/DVN/HMAYVV) as an example. Which would you trust? Why?

### Suggested Tasks:

1. Optional pre-class readings
   1. **Undergraduate students** read “[The TRUST Principles for digital repositories](https://www.nature.com/articles/s41597-020-0486-7)” (Lin et al., 2020).
   2. **Graduate students** should also read:
      1. “[Developing Criteria to Establish Trusted Digital Repositories](https://datascience.codata.org/article/10.5334/dsj-2017-022/)” (Fandeen, 2016).
      2. [Desirable Characteristics of Data Repositories for Federally Funded Research](https://doi.org/10.5479/10088/113528) (The National Science and Technology Council, 2022)
2. Using the provided slides to cover key concepts and discuss the following:
   1. What makes data useful vs. trustworthy?
      1. Make sure to emphasize how important it is to understand how the data was made and how it is documented and described.
   2. What makes the source of the data trustworthy and why is this important?
   3. Does this make data sets more or less reliable/trusted?
3. Activity for **undergrad and graduate students**, choose one:
   1. Visit [USDA Ag Data Commons](https://data.nal.usda.gov/). Can you locate information that satisfies each of the TRUST principles in at least one way? How difficult was the process?
   2. Choose a dataset in a database in your field. What information makes this data set more or less trustworthy? What about the database or the source of the data? (hint: sometimes you can find journal articles about biological databases.)
4. Activity for **graduate students**: Understand the purpose and expected content of a DMP
   1. Visit<https://dmptool.org/public_plans> and locate a featured plan to review.
   2. Discuss and compare DMPs. What sections were done well and easy to understand, what sections were difficult to follow? How different are plans across funders?

### References:

1. Faundeen J. Developing criteria to establish trusted digital repositories. (2017) *Data Science Journal*, 16:22-.
2. Lin D, Crabtree J, Dillo I, Downs RR, Edmunds R, Giaretta D, De Giusti M, L’Hours H, Hugo W, Jenkyns R, Khodiyar V. The TRUST Principles for digital repositories. (2020) *Scientific Data*, 7(1):1-5.
3. National Science and Technology Council (US). Desirable characteristics of data repositories for federally funded research. (2022) *Executive Office of the President of the United States*.
4. Castelli IE, Arismendi‐Arrieta DJ, Bhowmik A, Cekic‐Laskovic I, Clark S, Dominko R, Flores E, Flowers J, Ulvskov Frederiksen K, Friis J, Grimaud A. Data Management Plans: the Importance of Data Management in the BIG‐MAP Project. (2021) *Batteries & Supercaps*, 4(12):1803-12.
5. Fadlelmola FM, Zass L, Chaouch M, Samtal C, Ras V, Kumuthini J, Panji S, Mulder N. Data Management Plans in the genomics research revolution of Africa: Challenges and recommendations. (2021) *Journal of biomedical informatics*, 122:103900.
6. Gajbe SB, Tiwari A, Singh RK. Evaluation and analysis of data management plan tools: a parametric approach. (2021) *Information Processing & Management*, 58(3):102480.
7. Michener WK. Ten simple rules for creating a good data management plan. (2015) *PLoS computational biology*, 11(10):e1004525.
8. Wilkinson MD, Dumontier M, Aalbersberg IJ, Appleton G, Axton M, Baak A, Blomberg N, Boiten JW, da Silva Santos LB, Bourne PE, Bouwman J. The FAIR Guiding Principles for scientific data management and stewardship. (2016) *Scientific data*, 3(1):1-9.
9. Williams M, Bagwell J, Zozus MN. Data management plans: the missing perspective. (2017) *Journal of biomedical informatics*, 71:130-42.

### Resources

#### Standards and certifications for digital repositories to achieve TRUST:

- [CoreTrustSeal](https://www.coretrustseal.org/)
- [Trustworthy Repositories Audit & Certification: Criteria and Checklist](https://www.crl.edu/sites/default/files/d6/attachments/pages/trac_0.pdf)

1. <https://www.usgs.gov/office-of-science-quality-and-integrity/usgs-trusted-digital-repositories-tdr>

#### Data management and sharing plans

- <https://dmptool.org/>
- <https://www.nal.usda.gov/data/data-management-plan-guidance>
- <https://www.nsf.gov/bfa/dias/policy/dmp.jsp>
- https://dataoneorg.github.io/Education/bp_step/plan/
- https://rdaf.nist.gov/TopLevelPage
- <https://www.uvu.edu/osp/docs/nsf-data-management-plan-instructions-and-template1.pdf>
- <https://www.usgs.gov/data-management/planning>
- https://sharing.nih.gov/data-management-and-sharing-policy/planning-and-budgeting-DMS/writing-a-data-management-and-sharing-plan

## Lesson Plan 5: Submitting data

**Lead:** Meg Staton, Leonore Reiser

**Primary audience(s):** UG, Masters, PhD

### Learning outcomes:

- Understand the importance of submitting the generated data to biological digital repositories
- Learn to identify appropriate repositories for their data types, and how to work with repositories to ensure data submission.
- Learn how to prepare data for submission and where to submit them.

### Summary of Tasks/Actions:

- Why should you submit your data?
  - Funding agencies and publishers mandate open access to the data that you generate and publish. The Office of Science and Technology Policy (OSTP) released a memorandum (also called Nelson memo) where they clearly state that “publications and their supporting data resulting from federally funded research” are made “publicly accessible without an embargo on their free and public release”.
  - Data that are publicly accessible can be reused in new research project, increasing, therefore, their potential value. In addition, publications with open access data are cited more. Thus, submitting data to biological repositories in a way that they are more findable, accessible, and reusable, will benefits your personal citation indexes and your career advancement.
- Where to submit your data
  - Before generating the data, work on your Data Management Plan (DMP) and identify the repositories where you will potentially submit your data, and the data formats they accept. See Lesson Plan 4 “Creating and sharing trustworthy data” to learn more on the DMP.
  - Biological data can be submitted to three types of repositories:
    - *Generalist repositories*: they accept any type and formats of data, even across disciplines. Examples are [are Figshare (](https://data.nal.usda.gov/)<https://knowledge.figshare.com/>[), Dryad (](https://data.nal.usda.gov/)<https://datadryad.org/stash>[), Zenodo (](https://data.nal.usda.gov/)<https://zenodo.org/>[)](https://data.nal.usda.gov/), and Ag Data Commons ( [https://data.nal.usda.gov/)](https://data.nal.usda.gov/), which was established to manage USDA funded research data. Data submission at generalist repositories is usually very simple, and most of them assign a Digital Object Identifier (DOI) to your dataset. Some of them have a version control system, which allows to track the updates, and may help you quantify data reuse (e.g., number of page views, number of data download, etc.). However, most generalistic repositories don’t require standardized data format and metadata during the submission, which can simplify the data submission process but limits future data reuse. In addition, some of these repositories may require a data submission fee.
    - *Generalist biological databases*: they are domain-specific generalist repositories. They accept specific types of biological data; for instance, the Sequence Read Archive (SRA; <https://www.ncbi.nlm.nih.gov/sra>) accepts high throughput sequencing data from all branches of life as well as metagenomic and environmental surveys, while ProteomeXchange (<https://www.proteomexchange.org/>) is specialized on proteomic data. The generalist biological DBs are usually stable archival resources. They assigned DOIs to your data, but also require the data and metadata to be formatted according to specific standards. For instance, SRA accepts genomic data such as BAM, SFF, and HDF5 formats and text formats such as FASTQ (<https://www.ncbi.nlm.nih.gov/sra/docs/submitformats/>). They validate and curate your data, even if not as much as community databases. However, they may only accept one type of data (e.g., you might need to submit genome sequence and gene annotation data in two separate generalist biological repositories). Also, formatting your data according to their guidelines can be tricky and time-consuming. They don’t accept all type of data (e.g., genetic maps, gene mapping results, etc.).
    - *Community databases*: they center on a single model organism (called Model Organism Databases or MODS), a set of related species, or taxonomic ranges (also called Clade-Oriented Databases or CODs). Many of them were were originally organized around reference genome resources, and are genetic, genomic, and breeding (GGB) database resources. Community databases make your data more findable and visible in your research community by integrating them with other related data thanks to the work of specialized biocurators. Community database may accept data not accepted somewhere else (e.g., genetic maps) but may not accept non peer-reviewed data. They may not assign DOIs to your data and may not implement reuse tracking systems.
  - How do you decide where to submit your data?
    - You can explore which databases are available that can fit your data through database registries, such as FAIRsharing (<https://fairsharing.org/>) and Re3Data (<https://www.re3data.org/>). Look at recommendations from publishers, data librarians, and biocurators. In general, if there is an archival repository specific for your data type (e.g., NCBI SRA for sequencing data), then use it. If there is a community database in your field, contact the management team and see if they accept your data or if they have any recommendations.
    - Many biological digital repository will ask you to select a data license when submitting your dataset. A license defines the terms of use of your data from someone else. To allow data reuse and interoperability, it is recommended that you select a standard and open license. The Creative Commons (CC) licences (<https://creativecommons.org/share-your-work/cclicenses/>) are a type of standard licenses, which differ based on the level of permission. For instance:
      - The Public Domain Dedication (CC0 1.0)license waives all possible copyright and related rights. If someone uses your data under this license, it is not required that they give you appropriate credit (e.g., citation).
      - Under the Attribution (CC-BY-4.0) license, others can copy, redistribute, and adapt your dataset provided that they give appropriate credit.
      - The Attribution Share-Alike (CC-BY-SA-4.0) license is similar to the CC-BY-4.0, but requires that the derivative works are distributed under this same licence.

For more information about data open license and databases, please visit this website (https://rdm.elixir-belgium.org/data_licences)

- - What if there is no recommended database for your data type?
    - You can contact the community databases in your field and ask for advice. They usually pull data from generalist biological repositories (or primary databases, but can accept some data types. For instance, MaizeGDB, a community database of corn GGB data, provides guidance on where to submit data that it does not accept for submission (e.g., <https://www.maizegdb.org/contribute_data#function>).
      - Suggested activity:What type of data does MaizeGDB accept? And What is does not accept?
    - If your data cannot be submitted to a generalist biological repository or a community DB, you should submit your data to a generalist data repository (e.g., Dryad, Zenodo, FigShare, etc.).
    - Some journals might also offer guidance (e.g., <https://www.springernature.com/gp/authors/research-data-policy/repositories-mandates/19540364>). However, they usually are not informed about specifi recommendations, especially if they have a broad focus. Also, some of them don’t have statements about data availability and do not enforce data sharing.
- Preparation for submitting your data
  - Once you choose where to submit your data, explore their requirements and format your data and metadata following standards. Different resources may have different requirements of data and metadata formats.
    - If there are community standards, please follow them. See for example the Minimum Information About Plant Phenotyping Experiments (MIAPPE; <https://www.miappe.org/>) for phenotypic data, or the GFF3 file for genome annotation (<https://www.ncbi.nlm.nih.gov/genbank/genomes_gff/>).
    - If there are no standards, here are some hints on good data formats vs bad data formats:
      - Avoid Excel when possible. Some information can be lost because of formatting errors.
      - Prefer text-based data format (e.g., ‘.txt’, ‘.csv’) to PDFs
  - Along with your data, you need to share metadata richly describing the data submitted. The type of metadata to share depends on the type of data; example of metadata to share are sample information (e.g., accession numbers, environmental conditions, etc.), data collection workflows (e.g., protocols, instruments), software versions, the R code you developed to analyze your data, etc.
  - If you submit your data to a community DB or a generalist biological repository that involve data curation, your data can go through a process of biocuration, which includes the following steps:
    - Data quality check and formatting
    - Standardization using controlled vocabularies and ontologies
    - Integration with other data type and information (e.g., literatures)

Suggested activity:

The following will be a series of exercises aimed at helping researchers identify appropriate data repositories and data formats for submission

1. **UG and Grad**: Think about your thesis or internship project:
   - 1. what data will your experiments generate or have generated?
     2. Where should your data go?
        1. Use FAIRsharing or RE3Data.org to search for a repository.
        2. Is there a community DB or generalist biological repository?
        3. If submitting to a specific journal can you identify specific recommendations/requirements?
     3. What standard metadata/data formats are available for your data?
        1. Identify publisher recommendations if they exist for reporting /inclusion in paper
        2. Identify (community) DB recommendations if they exist

### References:

- Nelson A. Memorandum for the heads of executive departments and agencies: Ensuring free, immediate, and equitable access to federally funded research. (2022)
- Colavizza G, Hrynaszkiewicz I, Staden I, Whitaker K, McGillivray B. The citation advantage of linking publications to research data. (2020) *PloS one*, 15(4):e0230416.

## Lesson Plan 6: How to use your library resources

**Lead:** Leyla Cabugos

**Primary audience(s):** UG, Masters, PhD, Professional

### Learning outcomes:

By the end of this lesson, you will be able to:

- Identify data services available at your institution or others.
- Find subject specialist librarians and curated research guides at your institution, or if not available, at other institutions.
- Access scholarly literature through your institution or as an independent researcher

### Summary of Tasks/Actions:

Lessons 3 and 5 covered how to define a research question that can be answered by searching biological databases, find and effectively navigate resources that address the research question, and obtain the data you have identified as relevant. Lesson 7 will survey sources of agricultural data. If you need support in these tasks, librarians and specialists are available to help you in various ways.

This module covers how services and resources available through your university or public library can help you find scholarly literature and data, and make your own research output findable, accessible, interoperable and reusable. Available services and resources vary among libraries, but this presentation will give you an idea of what may be available through your library, and introduce freely accessible alternatives where we have been able to identify them.

**Data services**

Many academic libraries provide a suite of data services and resources, which may be administered by data specialists and/or subject librarians.

Examples of data services available through libraries include

1. Providing consultations and/or instruction on:

- Data management planning
- Finding data
- Acquiring data
- Cleaning and transforming data
- Analyzing data
- Visualizing data
- Sharing and Depositing data
- Obtaining persistent identifiers for researchers and documents

1. Offering software licenses and workstations
2. Nurturing communities of practice, by moderating:

- Listservs
- Peer-to-peer mentorship and informal presentations

1. Managing an institutional repository for research data and scholarly outputs of university affiliates

**Librarian services**

Most university libraries have subject specialists librarians who maintain a broad awareness of information practices and products relevant to the disciplines they support. They are often involved in building the library’s collection of owned and licensed material in these areas. They also provide instruction on information research practice and tools through guest instruction sessions, for-credit courses, workshops, and individual or group consultations.

Library services and resources vary from campus to campus. Libraries coordinate and collaborate with other campus support centers, such as offices of research, centers for teaching and learning technologies, and information technology services, as well as academic programs. If you do not find a service through your library, try checking with these other entities at your institution. Researcher support may also be available through government administered libraries (such as the National Agricultural Library), GBD’s, and cooperative extension programs.

Consultations and/or instruction on:

- Translating your research question into a search strategy that meets your goals and available resources (including considerations of scope, comprehensiveness, and replicability)
- Selecting and navigating information sources
- Documenting your searches and source material
- Accessing information resources within and beyond the library
- Evaluating your options for disseminating research findings

Subject librarians also curate collections of relevant resources to serve as a starting point for information research in the disciplines they support. These are often referred to as research guides, subject guides or Libguides (referring to a platform used in many libraries). The resources listed on research guides represent prominent examples of the resources available, but are not exhaustive, and subject librarians can help you identify the resource best suited to your particular research question.

Other lessons in this series will introduce collections of data that have either been gleaned from narrative articles, or deposited directly, and often processed to facilitate their manipulation and combination across sources. But since papers are still the primary way research is communicated, and the process of data curation is labor intensive, these are still an important source of data.

Thematic collections of scholarly literature are often highlighted on Research Guides under headers like Article Databases, or Subject Databases. These primarily contain records for peer reviewed articles, but may also contain conference proceedings, theses, news, datasets, patents, and other formats. Reading the description will give you a sense of the scope of the content, including the dates covered. Article Databases range from the specialized (such as CAB Abstracts for agricultural literature) to multidisciplinary (such as Web of Science).

Many of these databases are fee-based, and licensed by your campus or library for use by their campus community. Many public universities make computers available to anyone who can visit the building, so licensed databases can be accessed this way. Research guides also often include some resources that are available to anyone with an internet connection. Examples include the Agricola database, as well as PubMed. Note that while these tools facilitate the discovery of content relevant to your research, they vary in the degree to which they provide full text access to the articles themselves. We will discuss ways to access paywalled articles in another section of this lesson.

**Data in Archives**

Libraries and archives also partner with researchers to preserve records from notable research and breeding programs. Like literature, researcher archives can contain both contextual information and data that have yet to be made readily findable, accessible or reusable. These can include historical observations of weather, site conditions, organism phenotypes, and anything else that may be in a researcher’s file drawer. You can use finding aids to view the scope and contents of a collection, which may include correspondence, accounts of collection expeditions, notes from field trials and breeding programs. Detailed inventory and digitization of unique collections is completed as resources allow, and is often driven by their use in research.

**Publicly Available Library Services**

Researcher support is also available through government administered libraries such as the National Agricultural Library. The library is a program of the United States Department of Agriculture, and supports the work of employees of the USDA as well as serving the public.

Some search tools (such as article databases) are available to the public. These are often produced by a government agency or university. Examples include Agricola and Pubmed, which are free to search and provide free access to much of the content you may discover in these collections. There are also free-to-search databases like DeepDyve that provide individuals with subscription-based access to a large collection of articles.

Other access options include browser extensions like Open Access Button and Unpaywall, which locate legal and free copies of papers on the open internet. Such access is only possible when authors make their work available on their own website, in an institutional repository or other publicly accessible venue. These browser extensions streamline the process of locating free copies if they exist.

Government or Land Grant institutions typically make their resources (physical and electronic) available to the public, though this often requires the user to physically visit the library. For those who cannot physically visit the library, loans from these collections can sometimes be arranged through a university or public library.

Suggested Activities:

- **Data Services Activity**

Explore the data services available through your campus library, either by searching your library’s website or doing an internet search for data services and your university. Also note that you can find useful resources, such as tutorials, templates for data management plans, and curated data sources on the websites of other institutions. Below are links to several data services pages you can explore.

- <https://instr.iastate.libguides.com/dmp>
- <https://www.lib.purdue.edu/researchdata>
- <https://guides.lib.calpoly.edu/DPL/DataSkills>

- **Librarian Services Activity**

Use your library’s website to locate the subject specialist librarian whose focus most closely matches your research area. Locate the research guide closest to your topic of interest. What path did you take to find it? Record any resources relevant to your research. If you cannot find such a guide on your library’s website, explore the following example or use an internet search engine to find a library research guide on your topic of interest: <https://guides.library.ucdavis.edu/plant-sciences/plant-breeding>.
Libraries purchase or subscribe to many resources on behalf of their users (e.g. campus community). Libraries participate in lending networks that allow their users to access physical materials (or scans thereof) held at other libraries. This process is referred to as interlibrary loan. View the following example of how to request an interlibrary loan, then locate guidance on how to place such a request through your library. <https://lib.calpoly.edu/search-and-find/borrow-renew-request/interlibrary-loan/>

- **Archival Data Activity**Not all relevant data are compiled in a database. Librarians and data specialists can help you search for data within published literature, or within archives. Read the description of one of the archival collections for a plant breeder at the University of California, Davis, to get a sense of the unpublished resources available in library archives. <http://pdf.oac.cdlib.org/pdf/ucdavis/spcoll/d054_cuvh.pdf>

### References:

- Murray M, O'Donnell M, Laufersweiler M, Novak J, Rozum B, Thompson S. A survey of the state of research data services in 35 US academic libraries, or" Wow, what a sweeping question". (2019) *Research Ideas and Outcomes*, 5:e48809.
- Ruediger, D. An Interview with Susan Ivey, Director of the Research Facilitation Service at North Carolina State University. (2022) Ithaka S+R. <https://sr.ithaka.org/blog/the-librarys-role-in-facilitating-users-experiences-navigating-computing-and-data-services/>
- Stein LD. Integrating biological databases. (2003) *Nature Reviews Genetics*, 4(5):337-45.

## Lesson Plan 7: Databases for agriculture

**Lead:** Annarita Marrano

**Primary audience(s):** Ag UG, Masters, PhD

### Learning outcomes:

- Can understand and list which data are generated and used in agricultural research
- Can identify where genetic, genomic, and breeding (GGB) data can be found
- Can explain what community databases are and how they support agricultural research
- Can explain what model species are and why they are important for discoveries in agriculture
- Can talk about AgBioData and the main mission
- Can explain the challenges of GGB community databases and how they are addressing it
- Can explain how community databases promote equitable data in agricultural research

### Summary of Tasks/Actions:

- Agriculture refers to the use of crop plants and domestic animals for sustaining the global human population by providing food and other products (Harris and Fuller, 2014). There is a broad diversity of species and data types used in agriculture. For instance, researchers collect and correlate phenotypic observations to individual genomic and genetic variation to identify which candidate genes are involved in a specific trait of interest, such as yield and disease resistance (Varshney et al., 2020). Similarly, studying gene expression, the proteome, and metabolome under a specific environmental condition (e.g., pathogen attack, drought stress, etc.) helps address fundamental biological questions that can be translated into improved crops and livestocks or better field management (Pott et al. 2021). Other fields of investigation in agricultural research are biodiversity conservation and climate sustainability (Priyanka et al. 2021). The overall goal of all these research studies is to address the needs of the different stakeholders in agriculture (e.g., growers, consumers, etc.), especially under the unstable and constantly evolving scenario of climate change.
- Sharing and reusing high-quality agricultural research data can be crucial for new discoveries and improvements. Agricultural research data, including genetic, genomic, and breeding data, can be found in
  - *Generalist repositories*, such as AgDataCommins, Dryad, Zenodo, etc.
  - *Generalist Biological Databases*, such as NCBI, EMBL, and the Sequence Read Archive (SRA)
  - *Community databases*

For a more detailed explanation of the difference among these three categories, please look at Lesson Plan 1 “What is a biological digital repository?” and Lesson Plan 5 “Submitting Data”*.* This lesson plan will focus mostly on community databases (DBs), in particular GGB community DBs.

- Community DBs are curated repositories of data and knowledge of a particular species or a collection of closely related species. For instance, GGB community DBs maintain and curate genome assemblies and annotations, individual profiles of genetic variants, trait measurements, transcriptomic and pathways data, etc. The advantage of using a community DB is that you can find all the information you need on a specific species in one place, along with tools to analyze or visualize these data. For instance, you can do a BLAST of a DNA or protein sequence against the genome assemblies maintained at the community DB, or you can visualize which genes and transcripts map around or within a genomic region of interest with the JBrowse tool.
- Community DBs are built upon the detailed work of biocurators, who are experts in your research field. Also, community DBs maintained data that are not accepted by other generalist repositories, such as genetic maps or gene mapping results. Community DB curators integrate data so that you can access all the information available on a subject in one place. However, GGB community DBs do not accept every type of data, because of storage and curation limitations. Also, they usually curate literature data, which means that they might not accept not peer-reviewed data.
- In agricultural research, we can find community DBs of model species, crops, and livestock. Model organisms are non-human species largely used in research to answer fundamental biological questions (e.g, DNA repair mechanisms, a protein folding process and structure, etc.). The model organisms used in agricultural research are usually easy to grow (e.g., *Arabidopsis thaliana*) or to breed (e.g., Drosophila melanogaster, zebrafish) in the laboratory. Their study allows to make general conclusions for related species; for instance, many discoveries on flower gene expression and regulation in Arabidopsis are translated to the angiosperms, which include many important flowering crops. Maize is a crop used as a model organism to answer questions in fundamental and applied agricultural research; it has been a centerpiece for discoveries in genetics, cytogenetics, and genomics, as, for instance, the discovery of transposons by Barbara McClintock.
  - Suggested activity:
    - Read and discuss in the class the manuscript “*Barbara McClintock and the discovery of jumping genes*”, published on PNAS in 2012. <https://doi.org/10.1073/pnas.1219372109>
- The AgBioData consortium ( <https://www.agbiodata.org/>) involves over 40 GGB database resources, including community-DBs serving research communities working with model organisms, such as the Arabidopsis Information Resource - TAIR and MaizeGDB, crop species (e.g., Gramene, PeanutBase), animal (e.g., AnimalqtlDB) and insects (e.g., i5k Workspace@NAL). AgBioData also involves some generalist DBs such as the European Variation Archive or Ensembl. Established in 2015, AgBioData works with the agricultural research community and the member databases to define community-based standards for the acquisition and retrieval of GGB data, promoting the FAIR guidelines in agricultural research.
- Community-based standards are needed to facilitate and encourage data reuse in agriculture research, as well as improve data interoperability across GGB databases. Community DBs work together to address this need and find solutions for integrating data and knowledge from different resources. For instance, the general model organism database (GMOD) tool kit (<http://gmod.org>) is a collection of open source software tools for managing, visualising, storing, and disseminating genetic and genomic data. Chado is one of these tools, and is a relational database schema driven by ontologies. Databases that conform to this schema are perfectly interoperable. Other examples of tools that facilitate interoperability between community GGB databases are Tripal and Breeding API.
  - Tripal provides a common infrastructure framework based on existing biological standards, and the standardized object relational database schema, Chado. Tripal has a modular architecture, allowing developers to choose which building blocks to use for their biological data portal; one of these modules is *Tripal ElasticSearch*, which enables cross-site querying across databases. For instance, let’s say you are trying to find all the available data of *Junglas regia* (aka English walnut) in TreeGenes, the result page will show you data of *J. regia* available in other repositories that have implemented ElasticSearch (e.g., Citrus Genome Database). BrAPI is a web service API specification for communicating plant breeding data across different sources, as plant breeding DBs, genetic DBs, and end-user applications.
- The biggest challenge for community-databases is long-term financial support. Most of these database resources rely on public, limited funding that they use to cover all the costs related with data management and curation, mostly the salary of the database personnel. Once the project ends, if no other funding resources are available, a community-database risks to disappear along with the data it maintains, which can cause tremendous losses for the research community the database serves. Long-term financial sustainability of the database ecosystem is another core priority of the AgBioData consortium. To support the importance of community database for your research, please advocate for them, cite their release publication when publishing results based upon DB resources, and seek for collaboration and support with your community DB.
- Community DBs promote equitable research data; they provide global open-access data, breaking technological and financial barriers. For instance, data hosted in public repositories allows data reuse for new projects without even funding. In addition, community DBs provide free online tools to analyze and visualize data to everybody, connecting them across the globe.

### References:

- Peng FY, Hu Z, Yang RC. Genome‐Wide Comparative Analysis of Flowering‐Related Genes in Arabidopsis, Wheat, and Barley. (2015) *International journal of plant genomics*, (1):874361.
- Blümel M, Dally N, Jung C. Flowering time regulation in crops—what did we learn from Arabidopsis? (2015) *Current opinion in biotechnology*, 32:121-9.
- Huala E, Dickerman AW, Garcia-Hernandez M, Weems D, Reiser L, LaFond F, Hanley D, Kiphart D, Zhuang M, Huang W, Mueller LA. The Arabidopsis Information Resource (TAIR): a comprehensive database and web-based information retrieval, analysis, and visualization system for a model plant. (2001) *Nucleic acids research*, 29(1):102-5.
- Lawrence CJ, Dong Q, Polacco ML, Seigfried TE, Brendel V. MaizeGDB, the community database for maize genetics and genomics. (2004) *Nucleic acids research*, 32(suppl_1):D393-7.
- Ware D, Jaiswal P, Ni J, Pan X, Chang K, Clark K, Teytelman L, Schmidt S, Zhao W, Cartinhour S, McCouch S. Gramene: a resource for comparative grass genomics. (2002) *Nucleic acids research*, 30(1):103-5.
- Dash S, Cannon EK, Kalberer SR, Farmer AD, Cannon SB. Chapter 8—PeanutBase and other bioinformatic resources for peanut. (2016) *Peanuts*, 241-52.
- Hu ZL, Fritz ER, Reecy JM. AnimalQTLdb: a livestock QTL database tool set for positional QTL information mining and beyond. (2007) *Nucleic acids research*, 35(suppl_1):D604-9.
- Poelchau M, Childers C, Moore G, Tsavatapalli V, Evans J, Lee CY, Lin H, Lin JW, Hackett K. The i5k Workspace@ NAL—enabling genomic data access, visualization and curation of arthropod genomes. (2015) *Nucleic acids research*, 43(D1):D714-9.
- Cezard T, Cunningham F, Hunt SE, Koylass B, Kumar N, Saunders G, Shen A, Silva AF, Tsukanov K, Venkataraman S, Flicek P. The European Variation Archive: a FAIR resource of genomic variation for all species. (2022) *Nucleic Acids Research*, 50(D1):D1216-20.
- Martin FJ, Amode MR, Aneja A, Austine-Orimoloye O, Azov AG, Barnes I, Becker A, Bennett R, Berry A, Bhai J, Bhurji SK. Ensembl 2023. (2023) *Nucleic acids research*, 51(D1):D933-41.
- Papanicolaou A, Heckel DG. The GMOD Drupal bioinformatic server framework. (2010) *Bioinformatics*, 26(24):3119-24.
- Mungall CJ, Emmert DB, FlyBase Consortium. A Chado case study: an ontology-based modular schema for representing genome-associated biological information. (2007) *Bioinformatics*, 23(13):i337-46.
- Staton M, Cannon E, Sanderson LA, Wegrzyn J, Anderson T, Buehler S, Cobo-Simón I, Faaberg K, Grau E, Guignon V, Gunoskey J. Tripal, a community update after 10 years of supporting open source, standards-based genetic, genomic and breeding databases. (2021) *Briefings in Bioinformatics*, 22(6):bbab238.
- Selby P, Abbeloos R, Backlund JE, Basterrechea Salido M, Bauchet G, Benites-Alfaro OE, Birkett C, Calaminos VC, Carceller P, Cornut G, Vasques Costa B. BrAPI—an application programming interface for plant breeding applications. (2019) *Bioinformatics*, 35(20):4147-55.
